# Paired Associative Stimulation of the Auditory System: A Proof-Of-Principle Study

**DOI:** 10.1371/journal.pone.0027088

**Published:** 2011-11-02

**Authors:** Martin Schecklmann, Gregor Volberg, Gabriele Frank, Julia Hadersdorfer, Thomas Steffens, Nathan Weisz, Michael Landgrebe, Göran Hajak, Mark Greenlee, Joseph Classen, Berthold Langguth

**Affiliations:** 1 University of Regensburg, Department of Psychiatry and Psychotherapy, Regensburg, Germany; 2 University of Regensburg, Experimental Psychology, Regensburg, Germany; 3 University of Munich, Department of Neurology, Munich, Germany; 4 University of Regensburg, Department of Otorhinolaryngology, Regensburg, Germany; 5 University of Konstanz, Department of Psychology, Konstanz, Germany; 6 Hospital of Bamberg, Department of Psychiatry, Psychosomatics and Psychotherapy, Bamberg, Germany; 7 University of Leipzig, Department of Neurology, Leipzig, Germany; University of Salamanca- Mecial School, Spain

## Abstract

**Background:**

Paired associative stimulation (PAS) consisting of repeated application of transcranial magnetic stimulation (TMS) pulses and contingent exteroceptive stimuli has been shown to induce neuroplastic effects in the motor and somatosensory system. The objective was to investigate whether the auditory system can be modulated by PAS.

**Methods:**

Acoustic stimuli (4 kHz) were paired with TMS of the auditory cortex with intervals of either 45 ms (PAS(45 ms)) or 10 ms (PAS(10 ms)). Two-hundred paired stimuli were applied at 0.1 Hz and effects were compared with low frequency repetitive TMS (rTMS) at 0.1 Hz (200 stimuli) and 1 Hz (1000 stimuli) in eleven healthy students. Auditory cortex excitability was measured before and after the interventions by long latency auditory evoked potentials (AEPs) for the tone (4 kHz) used in the pairing, and a control tone (1 kHz) in a within subjects design.

**Results:**

Amplitudes of the N1-P2 complex were reduced for the 4 kHz tone after both PAS(45 ms) and PAS(10 ms), but not after the 0.1 Hz and 1 Hz rTMS protocols with more pronounced effects for PAS(45 ms). Similar, but less pronounced effects were observed for the 1 kHz control tone.

**Conclusion:**

These findings indicate that paired associative stimulation may induce tonotopically specific and also tone unspecific human auditory cortex plasticity.

## Introduction

Transcranial magnetic stimulation (TMS) is a noninvasive method for focal stimulation of superficial cortical areas. The magnetic field is produced by a changing electrical current in a coil that is placed over the skull at the area of interest [1]. The magnetic field passes the scull almost without any attenuation and causes action potentials via electro-magnetic induction. The rhythmic application of a series of TMS pulses (repetitive TMS, rTMS) has been shown to induce lasting inhibitory or facilitatory effects on excitability or function of particular brain sites. Most information about the effects of rTMS is obtained from studies of the motor cortex, as it is easy to assess the excitability of the cortico-spinal system by recording motor-evoked potentials from the target muscles [2]. Several lines of evidence suggest that rTMS can modulate synaptic plasticity via effects of long-term potentiation (LTP) or depression (LTD) [3]. On a neurobiological level changes of gene transcription (e.g., c-fos and brain-derived neurotrophic factor) and neurotransmitter release (e.g., glutamate and gamma amino-butyric acid) have been demonstrated [4]. Stimulation with low frequency rTMS (1 Hz and below) over the motor cortex has been shown to induce LTD-like effects [5]. The effects of single rTMS sessions normally last up to one hour [2], whereas repeated application of rTMS over several days has been shown to induce structural neuroplastic effects [6]. Based on its ability to induce effects on neuronal excitability that outlast the stimulation period, low frequency rTMS has been investigated as a treatment for many neuropsychiatric disorders characterized by focal hyper-excitability [5]. Thus, it has been shown that low frequency rTMS over temporal and temporo-parietal cortex can reduce tinnitus [7], [8] and auditory hallucinations [9]. Plewnia and colleagues conclude in their review, that “the response rate varies, the effect is predominantly moderate and the evidence for the stability of the effect is inconsistent [8]. Thus, more efficient stimulation protocols for tinnitus are needed [10].

Paired associative stimulation (PAS) [11] combines TMS pulses with a somatosensory stimulus at specific time intervals. It has been suggested that PAS induces associative or Hebbian long-term potentiation or depression of neuronal synapses via mechanisms of spike-timing dependent synaptic plasticity [12], [13]. If the somatosensory stimulus arrives at the cortex before TMS, facilitating effects on cortical excitability are induced, whereas if TMS is released before the cortical arrival of the somatosensory stimulus, depressant effects will follow. So far PAS has only been investigated for the somatosensory and motor system. Motor-evoked potentials [11], [13], [14] or somatosensory-evoked potentials [15], [16], [17], [18] were used as dependent variables to evaluate the effects of TMS together with the electrical stimulation of a peripheral nerve as paired exteroceptive stimulus (e.g., N. medianus). So far no study has investigated PAS of the auditory system.

Here we aimed to explore the effects of auditory cortex TMS paired with an auditory stimulus. In a within-subject design, four different protocols were applied in four separate sessions. In two PAS conditions an acoustic stimulus was paired with TMS of the auditory cortex with intervals of either 45 ms (PAS(45 ms)) or 10 ms (PAS(10 ms)), i.e., 45 ms or 10 ms after the acoustic stimulus onset the TMS pulse was applied. Two low frequency rTMS protocols at 0.1 Hz and 1 Hz without pure-tone auditory stimulation served as control conditions.

**Figure 1:**
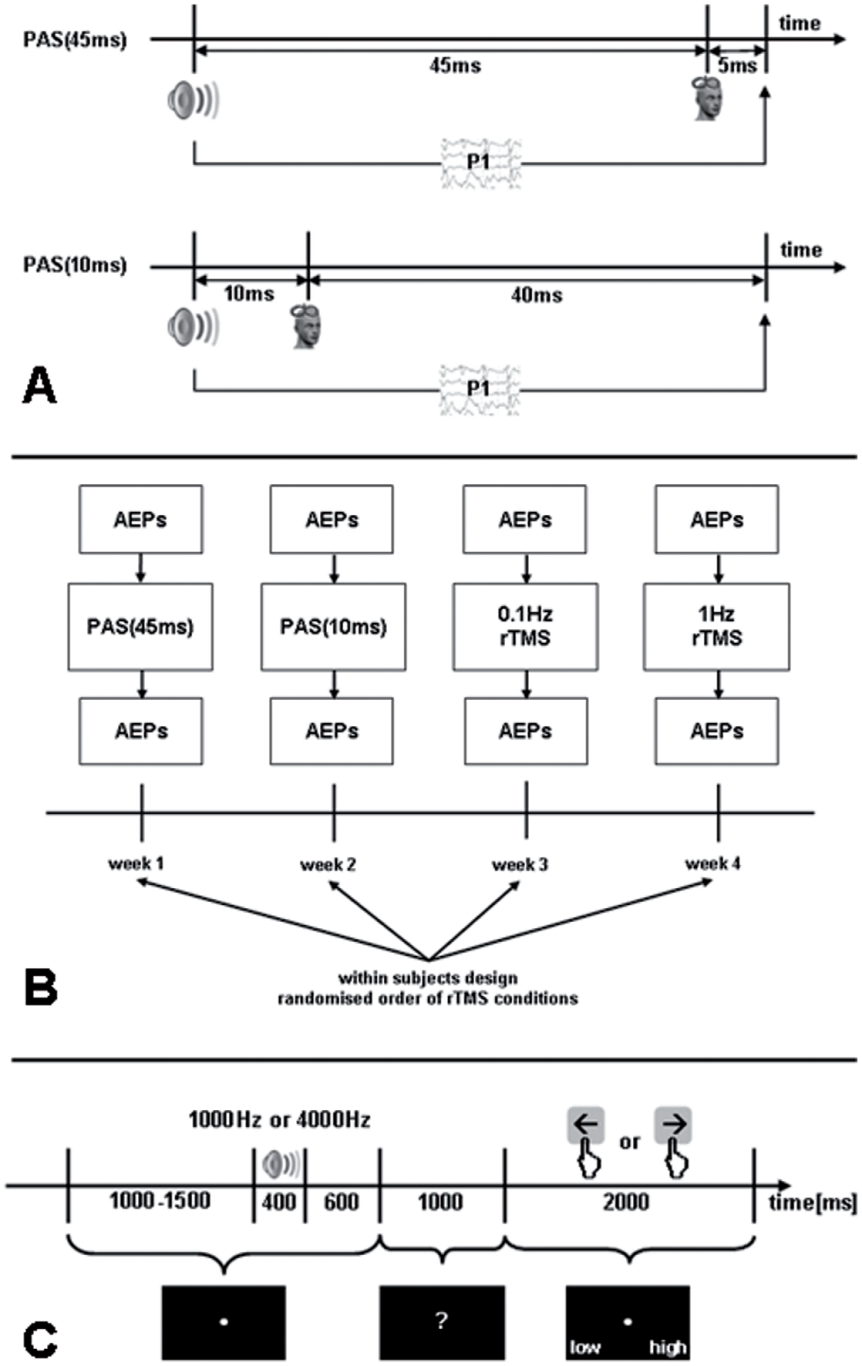
A) Single pulses of paired associative stimulation conditions (PAS(45 ms), PAS(10 ms)). P1 reflects the onset of cortical processing of the auditory stimulus in secondary auditory cortex. Thus, for both PAS conditions cortical processing starts after the TMS stimulus with the PAS(45 ms) being more close to the P1 than the PAS(10 ms). Therefore both conditions are considered inhibitory with a more pronounced inhibition for the PAS(45 ms). B) Study design (AEPs = acoustic evoked potentials; TMS =  transcranial magnetic stimulation; PAS = paired associative stimulation). C) Protocol of the measurement of auditory evoked potentials.

The design of the present study was drafted to measure long latency acoustic evoked potentials (AEPs) with origin in primary and secondary auditory cortex [19] which can be subsumed under the P1-N1-P2 complex with latencies of 50 ms (P1), 100 ms (N1), and 200 ms (P2). With 50 ms after stimulus onset the neuronal activity underlying the P1-N1-P2 complex starts for both PAS conditions after the TMS pulse (45 ms or 10 ms after stimulus onset). Therefore we expected after both PAS conditions amplitude reductions of the P1-N1-P2 complex. As a precise timing between cortical processing of the acoustic stimulus and TMS pulse is critical for spike-timing dependent plastic processes we expected rather a more pronounced effect for the PAS(45 ms) than for the PAS(10 ms) condition (compare figure 1A). An additional open question is whether the PAS effects are tonotopically specific or if they would also influence the AEPs of a control tone.

## Methods

### Subjects

Twelve young healthy volunteers participated in the study. For technical reasons (failed EEG trigger recordings in one subject), only data from eleven subjects (age: 21.4±1.5, 19-24 years; 8/3 female/male; 9/2 right-/left-hander) could be included in the analyses. Excluding the two left-handers from the analysis did not change the results. Only subjects with no history or presence of severe and relevant somatic, neurologic, or mental disorders were included. Vision was normal or corrected to normal. None of the subjects had a hearing loss of more than 30 dB HL in any of the seven measured audiometric frequencies ranging from 125 Hz to 8 kHz (Madsen Midimate 622D; GN Otometrics, Denmark). The study was approved by the Ethics Committee of the University of Regensburg. All procedures involved were in accordance with the last revision of the Declaration of Helsinki. All participants gave written informed consent after a comprehensive explanation of the procedures.

### Study procedures

Each subject participated in four experimental sessions (figure 1B), in which they received four different TMS conditions (paired associative stimulation with an interval between acoustic stimulus onset and TMS pulse of 45 ms (PAS(45 ms)) and 10 ms (PAS(10 ms)); very low frequency repetitive stimulation (0.1 Hz); low frequency repetitive stimulation (1 Hz)). The interval between sessions was one week to exclude possible TMS after-effects [20], [21]. An analysis of variance indicated no significant differences between the baseline measurements of the four sessions (F = 1.104; df = 3,30; p = 0.363). The order of stimulation conditions was randomised between subjects. Before and after stimulation AEPs were recorded. Thus, each experimental session consisted of the placement of the EEG cap (up to 30 minutes), a pre TMS AEP recording (ca. 10 minutes), TMS (up to 35 minutes), and a second AEP recording immediately after TMS (figure 1B). Before the first experimental session, informed consent was obtained, audiometry was performed and the resting motor threshold (RMT) was determined. The EEG recordings and TMS treatment took place in an electrically and acoustically shielded chamber with an external power supply in the Department of Experimental Psychology of the University of Regensburg.

### TMS protocols

Pulses were delivered with a Medtronic system (Medtronic, USA) and a figure of eight coil (90 mm outer diameter). The intensity of the stimulation was expressed as a percentage of the maximum output of the stimulator (0-100%) and was adjusted according to the RMT. The RMT was measured by delivering single pulses at the optimal location over the left motor cortex and was defined as the lowest stimulation intensity needed to produce a visible hand muscle contraction in at least five out of ten trials [22]. Then the coil was positioned over the left auditory cortex by using a standard procedure based on the 10-20-EEG, i.e., from T3 2.5 cm upwards on the line between T3 and Cz and then 1.5 cm in the posterior direction perpendicular to the line T3-Cz [23]. The 1 Hz condition consisted of 1000 pulses at a frequency of 1 Hz (total duration 17 minutes). The other three conditions consisted of 200 pulses at a frequency of 0.1 Hz (total duration about 33 minutes). Stimulation intensity for all conditions was 100% RMT or at 60% of the maximum TMS device output intensity, when RMT exceeded 60% of the maximum TMS device output. For PAS(45 ms) and PAS(10 ms) each TMS pulse was paired with a tone (4 kHz, 400 ms, 60 dB SPL) delivered to the right ear via an ER3A insert earphone with foam ear tips (Etymotic Research, USA). Right-sided auditory stimulation is thought to be processed predominantly in the left auditory cortex [24]. The left ear was occluded with the ear tip of the left side of the earphone (minimum of 30 dB SPL external noise exclusion). The PAS intervals were based on the earliest peak latency of 50 ms for the latency of the P1-N1-P2 complex. Thus, the onset of the acoustic stimulus was either 45 ms (PAS(45 ms)) or 10 ms (PAS(10 ms)) before the TMS pulse (figure 1A). With a typical delay of 50 ms which underlies the P1-N1-P2 complex the neuronal activity in the auditory cortex starts 5 ms (PAS(45 ms)) or 40 ms (PAS(10 ms)) after the TMS pulse. The 0.1 Hz condition served as a control condition where the same TMS pulses as in the PAS protocols were presented without a paired auditory stimulus. 1 Hz rTMS has been used as an additional control condition since it has been investigated in various preclinical [e.g., 6] and clinical [e.g., 7] studies. We used Presentation (Neurobehavioral Systems, USA) as the stimulation software on a common PC connected with the audiometer (analog channel) and with the TMS device (parallel port), to present the acoustic stimuli and to trigger the TMS device, respectively. Correct timing was approved via digitizing and measuring the auditory analog output signal and the TMS pulse in the same recording device.

### AEPs recording and measurement

AEPs were recorded from 62 equidistant electrodes that were mounted in an elastic cap (EasyCap, Germany) and were referenced to FCz during recording. Impedances were kept below 10 kΩ. The signals were digitized at a rate of 500 Hz (BrainAmp MR plus, Germany). Two different tones with frequencies of 1 kHz and 4 kHz respectively, a duration of 400 ms and an intensity of 60 dB HL were binaurally presented 50 times each in pseudo-randomised order. As we were interested in lateralized effects and as it is known that there are hemispheric differences for unilateral applied tones [24] we applied the tones binaurally to avoid this confoundation. The 4 kHz tone was identical with the paired tone of the PAS protocols. Before, during and after each auditory stimulus a white centrally located fixation point on a black background was constantly presented. The preceding interval varied between 1000 and 1500 ms; the succeeding interval was 600 ms long. Thereafter, a question mark prepared the subjects for the next screen showing the two possible answers “low” (1 kHz tone) and “high” (4 kHz tone) on the left and right lower corner of the monitor (figure 1C). Subjects were instructed to press the left or right arrow button of a common PC keyboard accordingly. The responses were intended to ensure attention to the tones during the whole task. Each complete session consisting of the EEG cap placement, pre TMS AEP recordings, TMS treatment and post TMS AEP recordings lasted approximately two hours. We used Presentation (Neurobehavioral Systems, USA) as the stimulation software on a common PC connected with a keyboard, a screen, and the audiometer, to record the manual responses, and to present the visual and the acoustic stimuli, respectively.

### Data analyses

After recording, the EEG data were filtered with a high-pass FIR filter of 0.4 Hz and segmented into epochs of 4 s centered at the tone onset. All epochs of one subject were concatenated over all conditions. The data were then subjected to an infomax independent component analysis in order to identify artefact components. Main sources of artefacts were eye blinks, eye movements, mains hum, and high muscle tonus. Artefact components were removed and the remaining components were back-projected to the EEG signal space. Finally, the data were visually inspected for any remaining artefacts. Thereafter the data was re-referenced to an average reference, the online-reference FCz was reconstructed, and electrodes with complete signal loss were interpolated. Re-referencing against linked mastoids did not change our results. For AEP analyses, sub-epochs of one second (200 ms before and 800 ms after the sound onset) were drawn from the data, where the 200 ms pre-stimulus-interval served as a baseline. Preprocessing and data visualization were done with the freely available MATLAB (Mathworks, USA) toolboxes EEGLAB [25] and FieldTrip [26].

The rationale for the selection of the dependent variable is described in the result section in detail. We calculated an analysis of variance (ANOVA) with three within-subjects factors, i.e., tone (4 kHz and 1 kHz), TMS stimulation condition (PAS(45 ms), PAS(10 ms), 0.1 Hz rTMS, and 1 Hz rTMS), and time (before and after TMS). To evaluate tone specific effects of the PAS conditions, as indicated by a significant threefold interaction, we calculated two-factorial ANOVAs with the factors TMS stimulation condition and time for the 4 kHz and the 1 kHz tone, respectively. To evaluate stimulation condition specific effects for the particular tones, we calculated two-tailed paired Student t-tests for the AEPs before and after TMS for all stimulation conditions. In addition, the significant pre-post contrasts were contrasted against each other corrected for pre TMS values (post TMS - pre TMS). Statistical analyses were done with SPSS 18.0.0 (SPSS, USA).

## Results

All eleven participants completed all experimental sessions. Side effects of TMS were rare. One single subject reported facial pain during the PAS(10 ms) condition. All subjects rated the tones during AEP recordings correctly as low or high tones in more than 98% of the trials. Thus, we assume that all subjects were highly attentive during the recordings.

### Plausibility of AEPs and definition of the dependent variable

**Figure 2:**
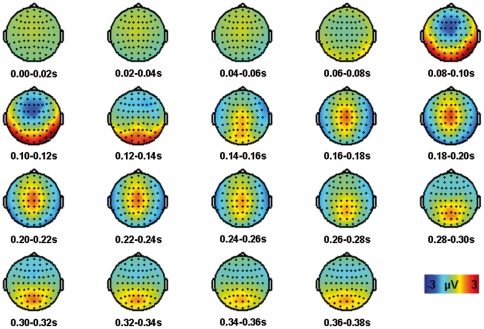
Topographies from 0 to 0.38s averaged in steps of 0.02s for the grand average of all pre stimulation conditions.

**Figure 3:**
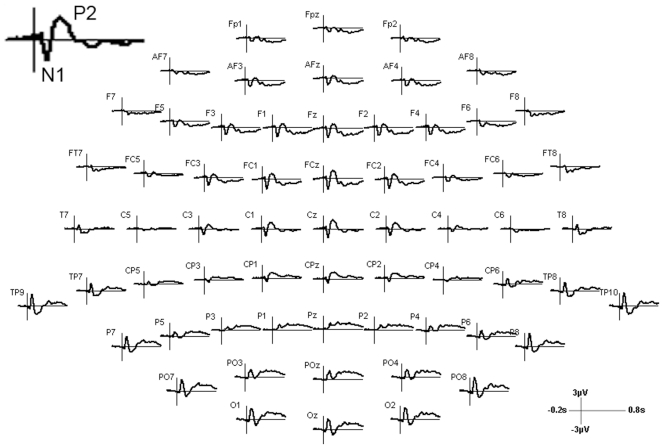
Trajectories of the grand average of all pre stimulation conditions for each electrode position.

We could clearly identify two typical peak components of the long latency auditory event-related potential (AEP): the N1 with a negative peak around 100 ms after sound onset with fronto-central topography and the P2 with a positive maximum around 200 ms with central topography (figure 2, figure 3). Inverse potentials (positive peaks at 100 ms and negative peaks at 200 ms) at left and right temporo-occipital electrodes mirrored the inversed topography of central N1 and P2 representing the different end of the auditory cortex dipoles. These posterior potentials did not exhibit any laterality effect. The P1 could not be unambiguously identified. The T-complex consists of long latency AEPs with peak latencies comparable to the P1-N1-P2 complex and dipoles in auditory areas; however, the center of the topography is located at temporal electrodes [27]. The T-complex with a positive peak around 100 ms (Ta) and a negative peak around 150 ms (Tb) was only observed at the right electrode T8 ipsilateral to the auditory stimulation, but not at the expected contralateral T7 [24]. We also did not find later potentials such as the N2 and P3. This might be due to the fact that these components are associated with attentional processes or particular tasks [19] mainly elicited by oddball paradigms [28].

Thus, our analyses were concentrated on the N1-P2 complex. Both components have their origin in the primary and/or secondary auditory cortex, which was the target region of our TMS treatment [19], [24]. Thus, for statistical analyses, we visually inspected the time line and the topography of these components and chose for the N1 a time interval from 75 to 125 ms and for the P2 from 150 to 250 ms at fronto-central electrodes (F3, F1, Fz, F2, F4, FC3, FC1, FCz, FC2, FC4, C3, C1, Cz, C2, C4, CP1, CPz, CP2). We averaged the two potentials over the electrodes and the chosen time windows and subsequently calculated the difference of the averaged amplitudes of both potentials.

As PAS effects are based on a strict timing between the TMS pulse and the acoustic stimulus it would have been interesting to conduct correlation analyses between individual P1 peak times with the PAS effects as it is known that P1 peaks vary between subjects within 40–80 ms after stimulus onset. However, as we did not find a clear P1 peak we abstained from such analyses and suggest this kind of analyses for future studies.

### Effects of TMS stimulation

**Figure 4:**
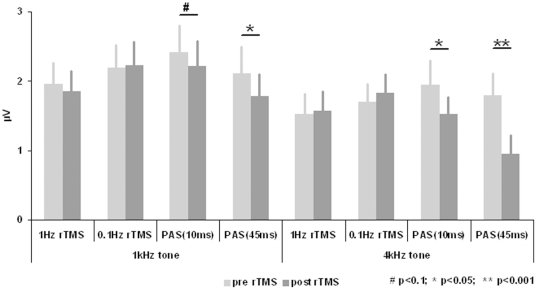
Amplitudes of the N1-P2 complex (difference of the amplitudes of both components) (mean±se). N1 amplitudes were averaged for the time interval from 75 to 125 ms and P2 amplitudes from 150 to 250 ms at fronto-central electrodes (F3, F1, Fz, F2, F4, FC3, FC1, FCz, FC2, FC4, C3, C1, Cz, C2, C4, CP1, CPz, CP2).

Results are depicted in figure 4. We found a “tone by TMS stimulation condition by time” interaction effect with a statistical trend (F = 2.820; df = 3,30; p = 0.056). Post hoc ANOVAs indicated a significant “stimulation condition by time” interaction effect for the 4 kHz (F = 5.454; df = 3,30; p = 0.004), but not for the 1 kHz tone (F = 1.084; df = 3,30; p = 0.371). Post hoc t-test for the 4 kHz tone indicated significant amplitude reductions for both PAS conditions, but not for the control conditions (PAS(45 ms): p<0.001; PAS(10 ms): p = 0.028; 0.1 Hz: p = 0.599; 1 Hz: p = 0.803). Effect size was high for the PAS(45 ms) condition (d = 1.506) and moderately high for the PAS(10 ms) condition (d = 0.775). Exploratory t-tests for the 1 kHz tone indicated significant or near significant amplitude reductions for both PAS conditions, but not for the control conditions (PAS(45 ms): p = 0.041; PAS(10 ms): p = 0.067; 0.1 Hz: p = 0.835; 1 Hz: p = 0.574). Effect sizes were moderately high for the PAS(45 ms) (d = 0.704) and the PAS(10 ms) condition (d = 0.616). Pre TMS corrected contrasts (post TMS - pre TMS) between the (significant) PAS conditions indicate that the amplitude reduction was greater for the 4 kHz tone in the PAS(45 ms) condition in contrast to the 4 kHz tone in the PAS(10 ms) condition (p = 0.019) and in contrast to the 1 kHz tone of the PAS(45 ms) condition (p = 0.025). Amplitude reduction for the 1 kHz tone of the PAS(10 ms) was not significantly different in contrast to the 4 kHz tone of the PAS(10 ms) condition (p = 0.154) and in contrast to the 1 kHz tone of the PAS(45 ms) condition (p = 0.334). In conclusion, primary analyses indicated tone specific effects of the PAS conditions with more pronounced effects for PAS(45 ms). Contrary to our expectations, the amplitude of the AEP to the 1 Hz tone was also reduced in both PAS conditions, although with less magnitude.

## Discussion

Our results demonstrate for the first time the applicability and the effectiveness of paired associative stimulation (PAS) over auditory cortex. It could be demonstrated that pairing TMS with an auditory stimulus modulates the excitability of the auditory cortex. Both PAS conditions resulted in a reduction of the N1-P2 amplitudes whereas the rTMS control conditions (0.1 Hz and 1 Hz) without paired auditory stimulation showed no effects. The effect sizes were more pronounced for PAS(45 ms) as compared to PAS(10 ms) and these were greater for the paired 4 kHz tone than for the 1 kHz control tone. This is in accordance with our expectations as on the one hand the timing between cortical processing of the tone and TMS pulse is more tightly synchronised for the PAS(45 ms) as for the PAS(10 ms) condition (figure 1A) and on the other hand TMS was paired with the 4 kHz tone. The neuroplastic mechanism of PAS effects is considered to be spike timing dependent plasticity, i.e., synaptic connections are strengthened or weakened by two tightly synchronised inputs to the synapse [12], [13]. The lack of a clear temporal specificity (significant effects for PAS(45 ms) and also for PAS(10 ms)) suggests that the exact timing of the arrival of stimulus-triggered activity in the auditory cortex might vary from trial to trial by as much as 30 ms. The tone specific effect is in accordance with the reports of topographical specificity of PAS over motor cortex, where PAS effects have been specifically demonstrated for the stimulation of corresponding muscle and motor cortex sites [11], [29]. However notably in contrast to the results from the motor system we observed also a tonotopically unspecific effect in addition to the tonotopically specific effect, since exploratory analyses also indicated an amplitude reaction for the 1 kHz control tone after the PAS protocols. This lack of tone specificity might suggest that TMS paired with a pure tone affects neural responding in regions that are not tonotopically organized (e.g., parabelt region of auditory cortex).

The latter tone non-specific effect is of considerable relevance for the potential therapeutic application of PAS in the treatment of tinnitus or other forms of auditory phantom perception as the exact matching of a tone to the perceived auditory phenomenon is frequently difficult.

Even if our results provide the proof-of-principle for applicability of the PAS paradigm on the auditory system, several open questions remain to be resolved by further investigations. We studied only immediate effects in a sample of young healthy subjects after one single TMS session. Thus, it would be of interest to determine how long the effects last, if similar effects can be obtained in clinical samples and if effects could be increased by additional sessions over several days. As both rTMS control conditions (the direct control condition 0.1 Hz and the clinically approved general control condition 1 Hz) showed no immediate effects, we consider the present PAS effects as boosting effects that might exceed clinical effects of low frequency stimulation protocols.

The design of the present study was drafted to measure the long latency AEPs. We found clear N1 and P2 amplitudes, but no valid P1. It should be taken into account that we based our considerations on the timing between acoustic stimulus and TMS pulse on the P1 latency. However, as the P1 is one part of the P1-N1-P2 complex N1 and P2 amplitudes should be representative also for P1 effects. In addition, it would be of interest to investigate middle latency potentials such as the Pa which is generated in the primary auditory cortex and which is considered to represent the earliest arrival of acoustic information in auditory cortex with a latency of 25-30 ms [30], [31], [32], [33]. Thus, for PAS(45 ms) the Pa would be generated before the TMS pulse and for PAS(10 ms) after the TMS pulse. Thus, one would expect an increase of Pa after PAS(45 ms) and a reduction after PAS(10 ms) if the TMS pulse has a direct effect on the auditory cortex, which is currently still a matter of debate [34], [35]. Our findings suggest that TMS has a direct effect on the secondary auditory cortex since both PAS conditions reduced the amplitude of the N1-P2 complex, which starts after about 50 ms and is generated in the secondary auditory cortex. Thus, systematic investigations of different intervals between the acoustic stimulus and TMS pulse and of different AEPs would reveal information about the most effective PAS protocol and about the question if PAS acts on the level of primary or secondary auditory cortex or both. For this question it would be also of considerable interest to replicate the present findings with functional imaging methods.

Another open question and a potential confounding factor in the present study is the acoustic stimulation inherent to every TMS application. Every TMS pulse is accompanied by a characteristic “click” sound. This TMS click is processed in the auditory cortex after the TMS pulse, i.e., every single TMS pulse might per se act as inhibiting paired associative stimulation [7], [36]. We attempted to shield the participants' ears from these clicks. But even with special earplugs, complete shielding could not be achieved. However if the “click” produced by the TMS pulse were relevant as an inhibitory paired acoustic stimulus (click is cortically processed after the TMS pulse), one would expect to observe this effect in the 0.1 Hz control condition. However, after 0.1 Hz we observed no amplitude reductions. Thus, the clicks produced by the TMS coil cannot be responsible for the amplitude reductions after both PAS conditions. However, interference between the TMS related clicks and the PAS effect cannot be excluded and should be investigated in future studies.

A further possible explanation for the inhibitory effects of both PAS conditions may be the length of the auditory stimuli presented. The duration of 400 ms for the tones presented is much longer when compared to somatosensory PAS protocols where the duration of hand nerve stimulation is in the range of microseconds. Even if the onsets of the auditory stimulus and the TMS pulse were precisely timed, the relative long duration of the auditory stimulus may have contributed to the inhibitory effect of both PAS conditions. In the somatosensory system, both active muscle innervation and attention focussing on the muscle without muscle contraction have an influence on PAS effects [37], [38]. In this pilot study we chose the duration of the tone according to standard protocols for auditory evoked potentials. Further studies will be needed to evaluate the role of the duration of the auditory stimulus.

Also habituation effects may be a possible explanation for our finding of decreased amplitude after PAS. The 4 kHz tone is presented 50 times for the AEP measurement before and 50 times after the TMS, which is comparable to the presentation rate of the 1 kHz tone. During the PAS conditions the 4 kHz tone is presented another 200 times. This high number of presentations may induce habituation effects resulting in diminished amplitudes. However, since habituation cannot explain the differential effects of PAS(45 ms) and PAS(10 ms) on the 4 kHz tone and the PAS effects on the 1 kHz control tone, pure habituation effects do not provide a sufficient explanation for our results. We cannot exclude an interaction between habituation effects and PAS. Thus one could speculate that habituation is influenced by the PAS conditions in different ways, i.e., the PAS(45 ms) facilitates habituation effects. Therefore future studies should include a further condition involving sham TMS associated with a tone, presentation of clicks without TMS, or TMS over non-auditory cortical areas. In addition, the time interval between the EEG measurements pre and post TMS should be held constant; since in the present study the 1 Hz control condition did not last as long as the other conditions. Furthermore, as the presentation rate of the control tone (only during the EEG measurement) was different from the number of presented PAS tones (during EEG and during PAS) future studies could prevent differential habituation effects specially related to the PAS tone by including one condition for which the control tone is paired with the TMS pulse outside a time window of spike timing dependent plasticity.

In conclusion, this proof-of-principle study is the first one showing PAS effects of auditory cortex. We found long latency AEP amplitude reductions specifically associated with the tone that was associated with the PAS conditions and the PAS condition with close timing between acoustic stimulus and TMS pulse. Exploratory analyses also indicated non-specific effects as indicated by amplitude reductions for AEPs of the control tone. TMS with paired-associated stimulation reduced the AEP amplitude whereas rTMS without paired auditory stimulation did not. This finding suggests that PAS might prove to be a more effective treatment of tinnitus or other disorders with acoustic phantom perception than rTMS alone. Still, open questions remain that are related to the effects of different PAS intervals and different AEPs, to measuring effects by other imaging methods, to lateralised effects - which showed an insufficient signal-to-noise ratio in the present study - and to the influence of the duration of the auditory stimulus.
